# Study on the mechanism of action of colchicine in the treatment of coronary artery disease based on network pharmacology and molecular docking technology

**DOI:** 10.3389/fphar.2023.1147360

**Published:** 2023-06-19

**Authors:** Yunfeng Yu, Manli Zhou, Xi Long, Shuang Yin, Gang Hu, Xinyu Yang, Weixiong Jian, Rong Yu

**Affiliations:** ^1^ College of Chinese Medicine, Hunan University of Chinese Medicine, Changsha, Hunan, China; ^2^ The First Affiliated Hospital of Hunan University of Chinese Medicine, Changsha, Hunan, China; ^3^ Key Laboratory of Chinese Medicine Diagnostics in Hunan Province, Hunan University of Chinese Medicine, Changsha, Hunan, China

**Keywords:** colchicine, coronary artery disease, network pharmacology, molecular docking, mechanism of action

## Abstract

**Objective:** This is the first study to explore the mechanism of colchicine in treating coronary artery disease using network pharmacology and molecular docking technology, aiming to predict the key targets and main approaches of colchicine in treating coronary artery disease. It is expected to provide new ideas for research on disease mechanism and drug development.

**Methods:** Traditional Chinese Medicine Systems Pharmacology Database and Analysis Platform (TCMSP), Swiss Target Prediction and PharmMapper databases were used to obtain drug targets. GeneCards, Online Mendelian Inheritance in Man (OMIM), Therapeutic Target Database (TTD), DrugBank and DisGeNET databases were utilized to gain disease targets. The intersection of the two was taken to access the intersection targets of colchicine for the treatment of coronary artery disease. The Sting database was employed to analyze the protein-protein interaction network. Gene Ontology (GO) functional enrichment analysis was performed using Webgestalt database. Reactom database was applied for Kyoto Encyclopedia of Genes and Genomes (KEGG) enrichment analysis. Molecular docking was simulated using AutoDock 4.2.6 and PyMOL2.4 software.

**Results:** A total of 70 intersecting targets of colchicine for the treatment of coronary artery disease were obtained, and there were interactions among 50 targets. GO functional enrichment analysis yielded 13 biological processes, 18 cellular components and 16 molecular functions. 549 signaling pathways were obtained by KEGG enrichment analysis. The molecular docking results of key targets were generally good.

**Conclusion:** Colchicine may treat coronary artery disease through targets such as Cytochrome c (CYCS), Myeloperoxidase (MPO) and Histone deacetylase 1 (HDAC1). The mechanism of action may be related to the cellular response to chemical stimulus and p75NTR-mediated negative regulation of cell cycle by SC1, which is valuable for further research exploration. However, this research still needs to be verified by experiments. Future research will explore new drugs for treating coronary artery disease from these targets.

## Introduction

Coronary artery disease (CAD) is a common heart disease characterized by insufficient blood and oxygen supply to the myocardium (Liang et al., 2021). It is caused by atherosclerosis and atherosclerotic plaque rupture leading to arterial occlusion or obstruction of smaller branches of coronary arteries by material dislodged from the plaque (Prasad and Elefteriades, 2021). Inflammatory activation and dysfunction of the coronary endothelium is a key link in the development of atherosclerosis, as well as being strongly associated with an increased risk of cardiovascular events (Medina-Leyte et al., 2021). Currently, CAD remains the leading cause of death worldwide (Lawton et al., 2022) and is a major source of global cardiovascular disease morbidity, mortality, and economic burden on health (Virani et al., 2021). Clinically, management of diet as well as lifestyle and usage of statins as well as antiplatelet drugs are mainly used as prophylactic treatment for CAD (Jia et al., 2020; Wong et al., 2022). Although antiplatelet aggregation, lipid regulation to stabilize plaque and control of risk factors have reduced the cardiovascular risk of patients to some extent (Imazio et al., 2020), the annual risk of major cardiovascular events is still as high as 3% (Amico et al., 2016; Eikelboom et al., 2017), so CAD is still in urgent need of intervention with novel therapy regimens. Colchicine has been reported to have potential application in cardiovascular disease (Tong et al., 2020), significantly reducing major adverse cardiovascular events (MACE) in patients with CAD (Nidorf et al., 2020; Kurup et al., 2021), and is expected to be a new drug for second-level prevention of CAD (Roubille and Tardif, 2020; Slomski, 2020). Colchicine was found to reduce hs-CRP at 0.36 mg/L and mean leukocytes at 371.75/L in patients with CAD (Sethuramalingam et al., 2021), implying that colchicine can reduce the inflammatory response in patients with CAD. Meta-analysis demonstrated that colchicine reduced MACE by approximately 27%–35%% (Yu et al., 2021; Khan and Lee, 2022a; Casula et al., 2022; Ma et al., 2022), and this benefit was independent of the clinical phenotype of CAD (Xia et al., 2021). In another study, colchicine was also shown to significantly reduce the incidence of stroke by 75% (Wudexi et al., 2021) and the incidence of ACS by 36% (Abrantes et al., 2021). This evidence suggests that colchicine has great potential in the treatment of CAD.

Colchicine, one of the oldest drugs still in use today, is derived from the dried bulb and seeds of the colchicum (Imazio and Nidorf, 2021). It is a low-cost drug with a wide range of anti-inflammatory properties and is currently used in diseases such as gout and familial mediterranean fever (Liuzzo and Patrono, 2020). Several recent meta-analyses have shown that colchicine significantly reduces MACE (Fiolet et al., 2021; Xiang et al., 2021; Andreis et al., 2022) and decreases the level of inflammatory markers in patients with CAD (Sethuramalingam et al., 2021), and the benefit of MACE still holds after percutaneous coronary intervention (Aw et al., 2022). Nevertheless, the mechanism of action of colchicine in the treatment of CAD has not been elucidated, and it is difficult to determine its long-term efficacy and safety.

Network pharmacology is to establish a network model of effective drug targets, involving emerging interdisciplinary subjects such as artificial intelligence and big data (Xia, 2017; Khan and Lee, 2022a). In the development of clinical trials of therapeutic drug candidates, the direction of their research can be guided by visualizing the “drug-targeted disease” network, which is of great significance for exploring the pathways of natural drug molecules related to the treatment of various diseases and other related research (Khan and Lee, 2022b). Therefore, this study aimed to predict the key targets and major pathways of colchicine for the treatment of CAD using network pharmacology and molecular docking techniques, and to explore the potential mechanisms of drug action.

## Methods

### Source of drug targets

The Traditional Chinese Medicine Systems Pharmacology Database and Analysis Platform (Zhang et al., 2016) (TCMSP, https://tcmsp-e.com/, accessed on 10 February 2022) was used to identify the targets of “Colchicine”. The Swiss Target Prediction database (Gfeller et al., 2013) (http://www.swisstargetprediction.ch/, accessed on 10 February 2022) was also used to predict the target of colchicine, and we use probability >0 as the screening condition to screen target. PharmMapper database (Wang et al., 2017) (http://www.lilab-ecust.cn/pharmmapper/, accessed on 10 February 2022) was utilized to predict the targets of colchicine, where we set up Generate Conformers as “yes”, Maximum Generated Conformations as “300” and Select Targets Set as “Druggable Pharmacophore Models (v2017, 16,159)”. The Uniprot database (Wang et al., 2021) (https://www.uniprot.org/, accessed on 10 February 2022) was used for matching the gene names of each target protein, and then the target genes from the three databases were pooled to obtain the drug targets of colchicine.

### Sources of disease targets

The GeneCards (Stelzer et al., 2016) (https://www.genecards.org/, accessed on 10 February 2022), OMIM (Amberger et al., 2015) (https://mirror.omim.org/, accessed on 10 February 2022), DrugBank (Wishart et al., 2018) (https://go.drugbank.com/, accessed on 10 February 2022), DisGeNET (Bauer-Mehren et al., 2010) (https://www.disgenet.org/, accessed on 10 February 2022), and TTD (Zhou et al., 2022) (http://db.idrblab.net/ttd/, accessed on 10 February 2022) databases were searched for “Coronary Artery Disease” to obtain the relevant disease targets. We used the default search function of the database, and included all CAD related targets provided by the database. The disease targets from the five databases were combined to obtain the disease targets for CAD. The drug targets were then intersected with the disease targets to obtain the intersecting targets, and the Venn diagram of the drug-disease intersecting targets was drawn using R-4.0.2-win software.

### Construction of protein-protein interaction network

The intersecting targets of drugs and diseases were imported into the String network platform (Szklarczyk et al., 2021) (https://string-db.org/, accessed on 10 February 2022). The protein type was set to “*Homo sapiens*”. The confidence level was set to >0.9. The free targets were deleted, and the protein-protein interaction network (PPI) was constructed. After importing the nodal relationship information into Cytoscape 3.8.0 software to calculate the degree, the key targets for colchicine in the treatment of CAD were predicted based on the degree (Long et al., 2020), and the PPI was mapped using Cyroscape3.8.0 software.

### Gene Ontology (GO) functional enrichment analysis

The drug-disease intersection targets were imported into the WebGestalt database (Liao et al., 2019) (http://www.webgestalt.org/, accessed on 10 February 2022). The restricted species was “*H. sapiens*”. The method was over characterization analysis. Using the genomic protein code as the reference set, GO functional enrichment analysis of biological process (BP), molecular function (MF) and cellular component (CC) was performed on the intersection targets.

### Kyoto Encyclopedia of Genes and Genomes (KEGG) pathway enrichment analysis

The drug-disease intersection targets were uploaded into the Reactome database (Griss et al., 2020) (https://reactome.org/, accessed on 10 February 2022). The species was qualified as “*H. sapiens*”. *p* < 0.05 and FDR < 0.05 were set. KEGG pathway enrichment analysis was performed on the intersecting targets. GO functional enrichment analysis and KEGG enrichment analysis were mapped using R-4.0.2-win software, and drug-target-pathway mesh maps were mapped using Cytoscape 3.8.0 software.

### Molecular docking of drug components-intersecting targets

The protein-data-bank (PDB) IDs of the intersecting targets were searched through the String network platform, and the corresponding protein structures were downloaded from the RCSB PDB database (https://www.rcsb.org/, accessed on 10 February 2022) based on the PDB IDs. If multiple PDB IDs exist for the same target, the best protein crystal structure was selected based on the root mean square deviation of the resolution. The proteins were processed using AutoDock4.2.6 (Goodsell et al., 2021) (https://autodock.scripps.edu/) and PyMOL2.4 (https://pymol.org/2/) (Rigsby and Parker, 2016) to remove small molecule ligands and all water molecules. The location of the docking box was determined based on the small molecule composition provided by the PBD database. If the PDB database did not provide the small molecule composition, the POCASA platform (http://g6altair.sci.hokudai.ac.jp/g6/service/pocasa/, accessed on 10 February 2022) was used to predict the position of the docking box. All parameters were default standard parameters of the software or platform. Molecular docking was carried out after hydrogenation and electron addition to the processed proteins, and the binding activity was judged by the docking score. The docked protein-ligand complexes were imported into the PLIP platform (https://plip-tool.biotec.tu-dresden.de/, accessed on 10 February 2022) for analysis, and molecular docking patterns were drawn in combination with PyMOL2.4 software.

**FIGURE 1:**
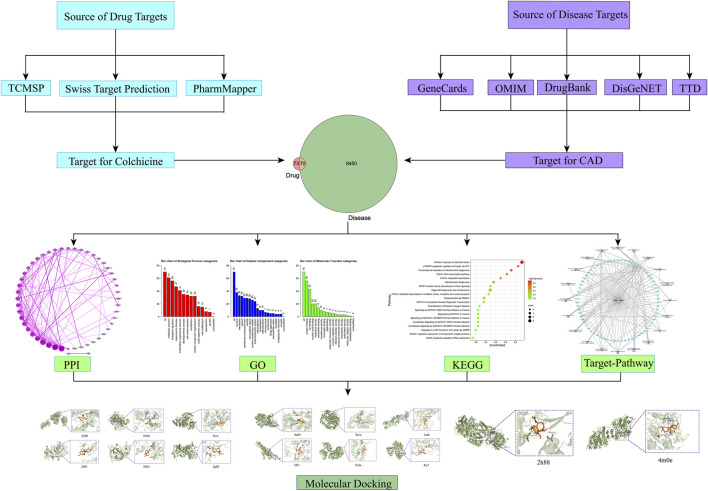
Flow chart of network pharmacology and molecular docking.

The workflow is demonstrated in Figure 1.

## Results

### Drug targets

The TCMSP database indicated that colchicine has a Mol ID of MOL013158, a bioavailability of 3.85% and a drug-like property of 0.78. A total of 143 potential targets of colchicine were obtained through TCMSP, Swiss Target Prediction and PharmMapper databases.

### Disease targets and intersection targets

**FIGURE 2:**
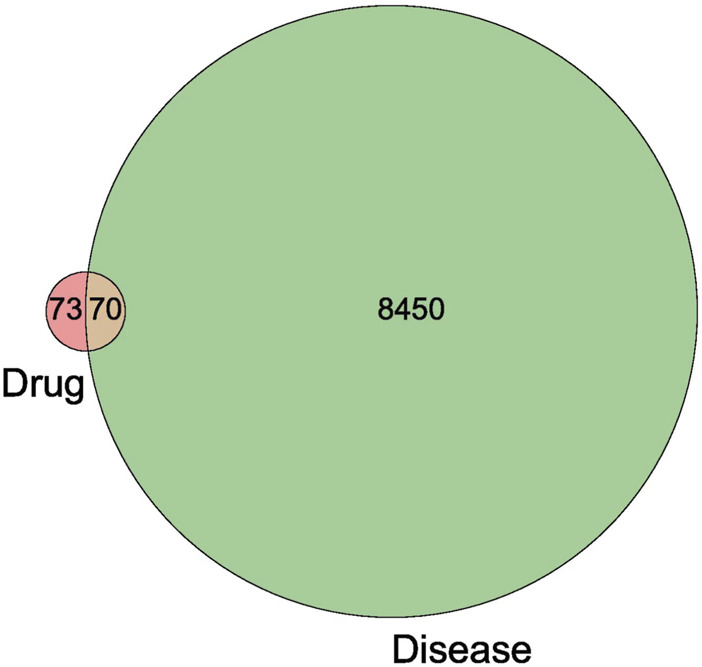
Venn diagram of the intersection target of colchicine and CAD.

A total of 8520 CAD-related targets were obtained through GeneCards, OMIM, DrugBank, DisGeNET and TTD databases. After intersecting them with drug targets, 70 targets of colchicine for the treatment of CAD were obtained (Figure 2).

### PPI

**FIGURE 3:**
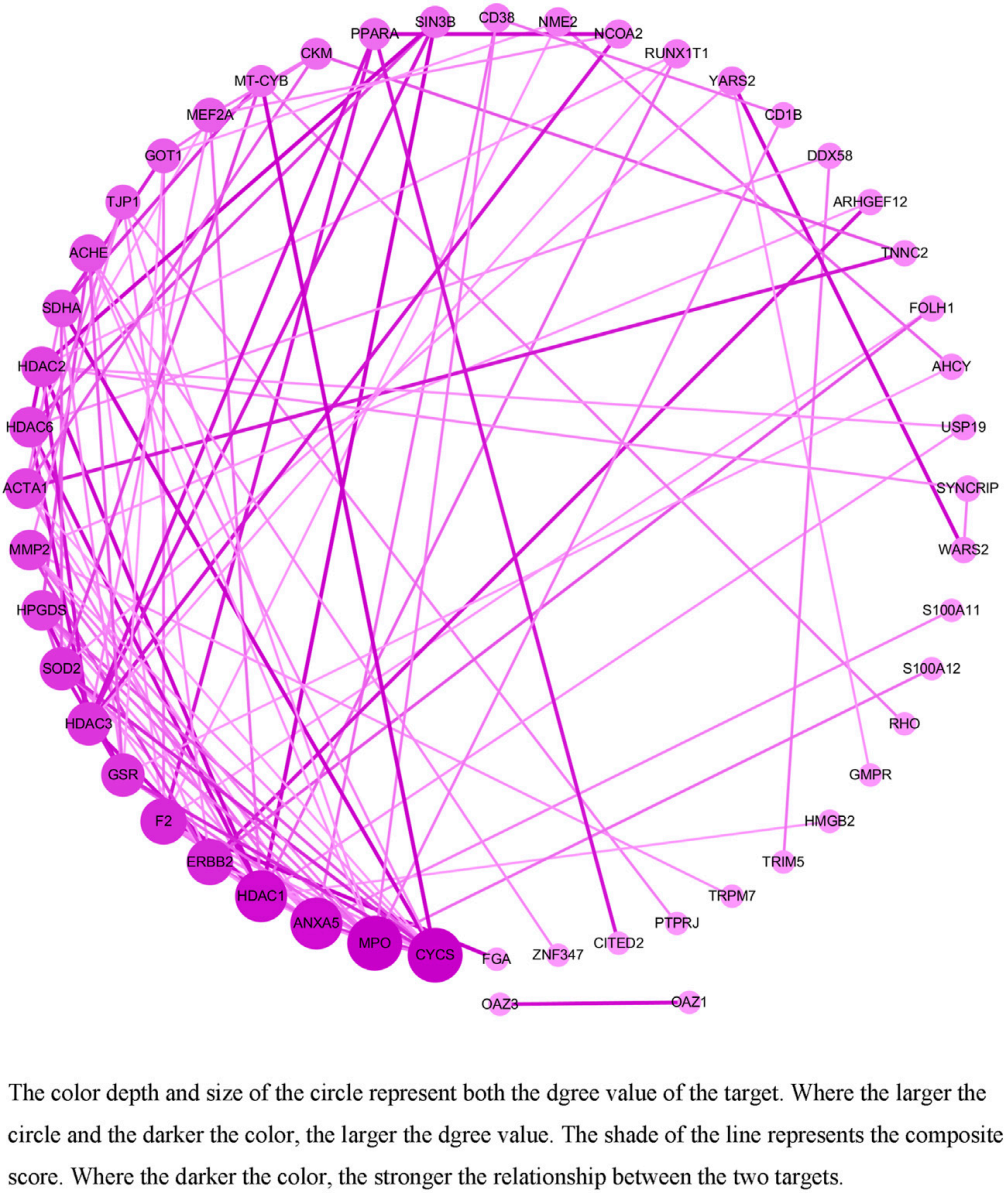
The PPI network of the target of colchicine in the treatment of CAD.

Seventy intersecting targets were imported into the String database, and the PPI network revealed that there were free targets for ACADS, ACKR1, ACR, BLVRA, CCL24, DAB1, EDC3, FERD3L, FLOT2, FUT8, KLK1, MYNN, PMP2, PRPS1, REPS1, RPA1, SNF8, TCEA3, TOX and TUBB1. And interactions were present for the remaining 50 targets, as shown in Figure 3. The node relationship information was imported into Cytoscape3.8.0 software to calculate the degree values, and the following results were displayed. 1) The degree values of Cytochrome c (CYCS), Myeloperoxidase (MPO), Annexin A5 (ANXA5) and Histone deacetylase 1 (HDAC1) were >10. 2) The degree values of Receptor tyrosine-protein kinase erbB-2 (ERBB2), Prothrombin (F2), Glutathione reductase (GSR), Histone deacetylase 3 (HDAC3), Superoxide Dismutase 2 (SOD2), Hematopoietic prostaglandin D synthase (HPGDS), 72 kDa type IV collagenase (MMP2), Actin, alpha skeletal muscle (ACTA1), Histone deacetylase 6 (HDAC6), Histone deacetylase 2 (HDAC2), Succinate Dehydrogenase Complex Flavoprotein Subunit A (SDHA) and Acetylcholine receptor subunit epsilon (ACHE) were >5. 3) The degree values of the remaining targets were ≤ 5.

### GO functional enrichment analysis

**FIGURE 4:**
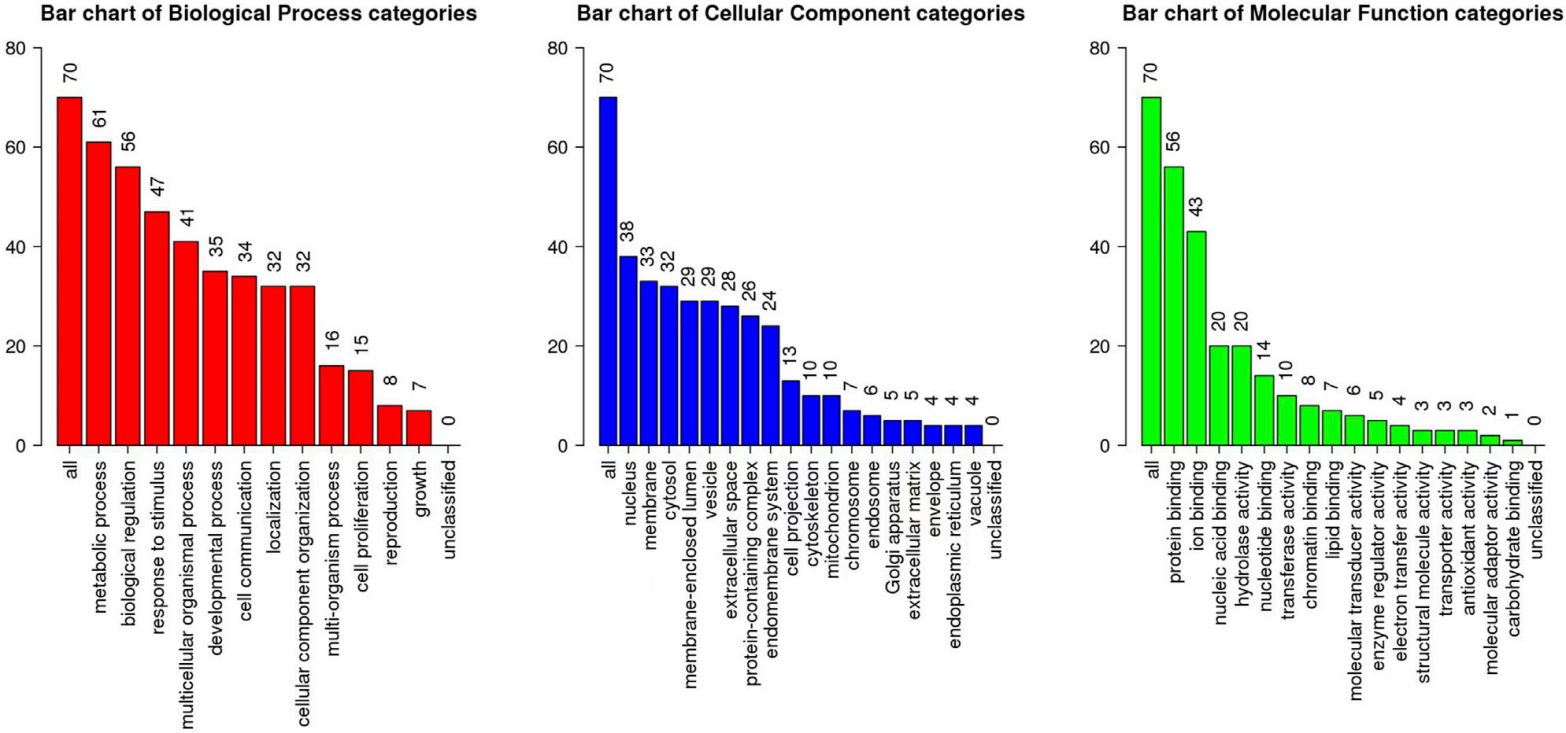
GO functional enrichment analysis of CAD targets in the treatment of colchicines.

The 70 intersecting targets were loaded into the Webgestalt database for GO functional enrichment analysis, and the results shown were as follows (Figure 4). 1) There were 13 BP for colchicine in the treatment of CAD, mainly involving metabolic processes, biological regulation, responses to stimuli, multicellular biological processes and growth processes. 2) There were 18 CC, mainly involving the nucleus, cell membrane, cytoplasm, membrane lumen and vesicles. 3) There were 16 molecular functions (MF), mainly related to protein binding, ion binding, nucleic acid binding, hydrolase activity and nucleotide binding, etc.

### KEGG pathway enrichment analysis

**FIGURE 5:**
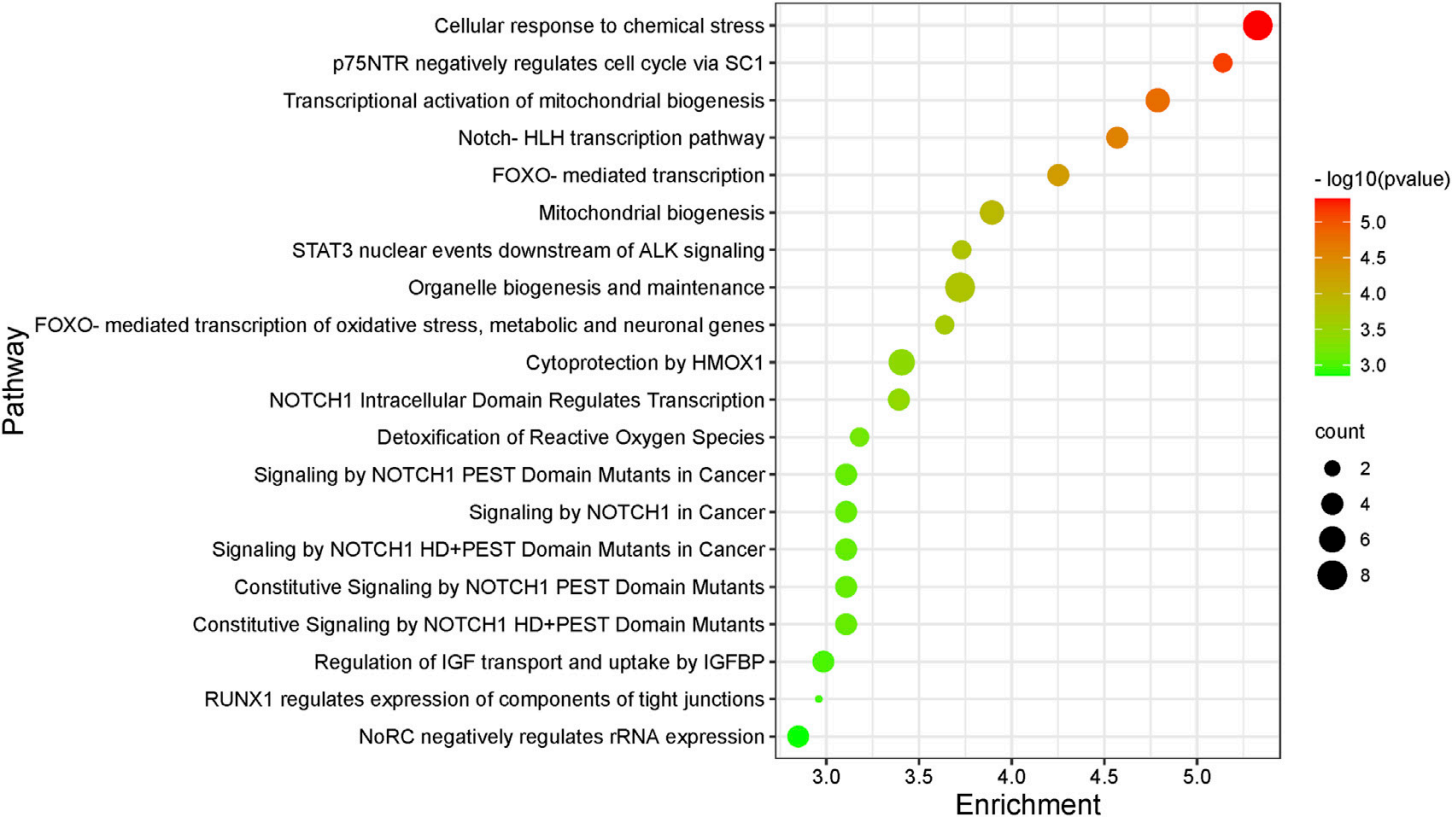
KEGG pathway enrichment analyses of the targets of colchicine in the treatment of CAD.

**FIGURE 6:**
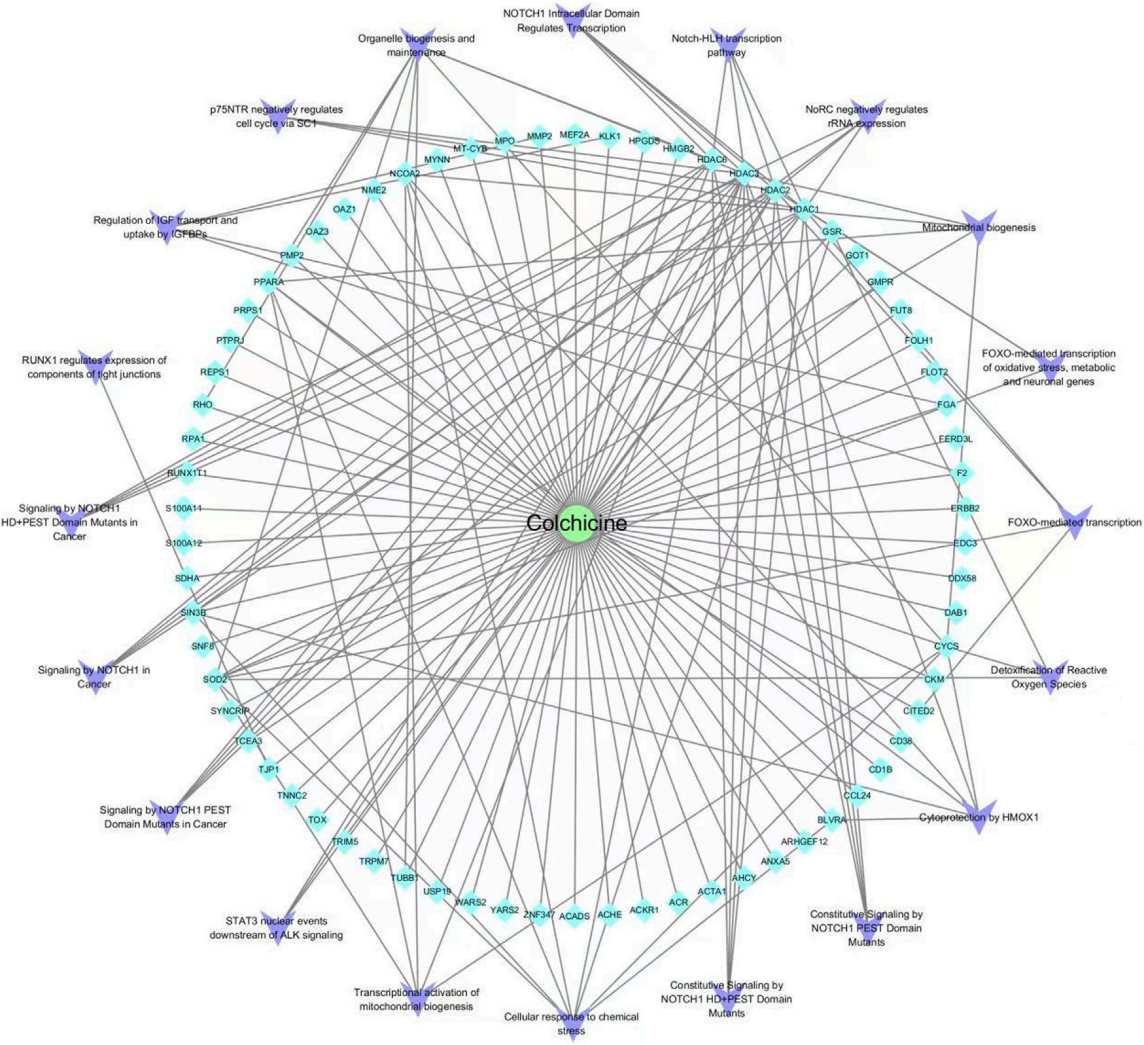
Drug-target-pathway network diagram of colchicine in the treatment of CAD.

70 intersecting targets were imported into the Reactom database for KEGG enrichment analysis. It revealed a total of 549 pathways for colchicine in the treatment of CAD, which involved cellular response to chemical stress, p75NTR-mediated SC1 negative regulation of cell cycle, transcriptional activation of mitochondrial biogenesis, Notch-HLH transcriptional pathway and FOXO-mediated transcription. The top 20 pathways are shown in Figure 5, and the reticulation of drugs, targets, and pathways is shown in Figure 6.

### Active ingredient-intersection target molecular docking

**FIGURE 7:**
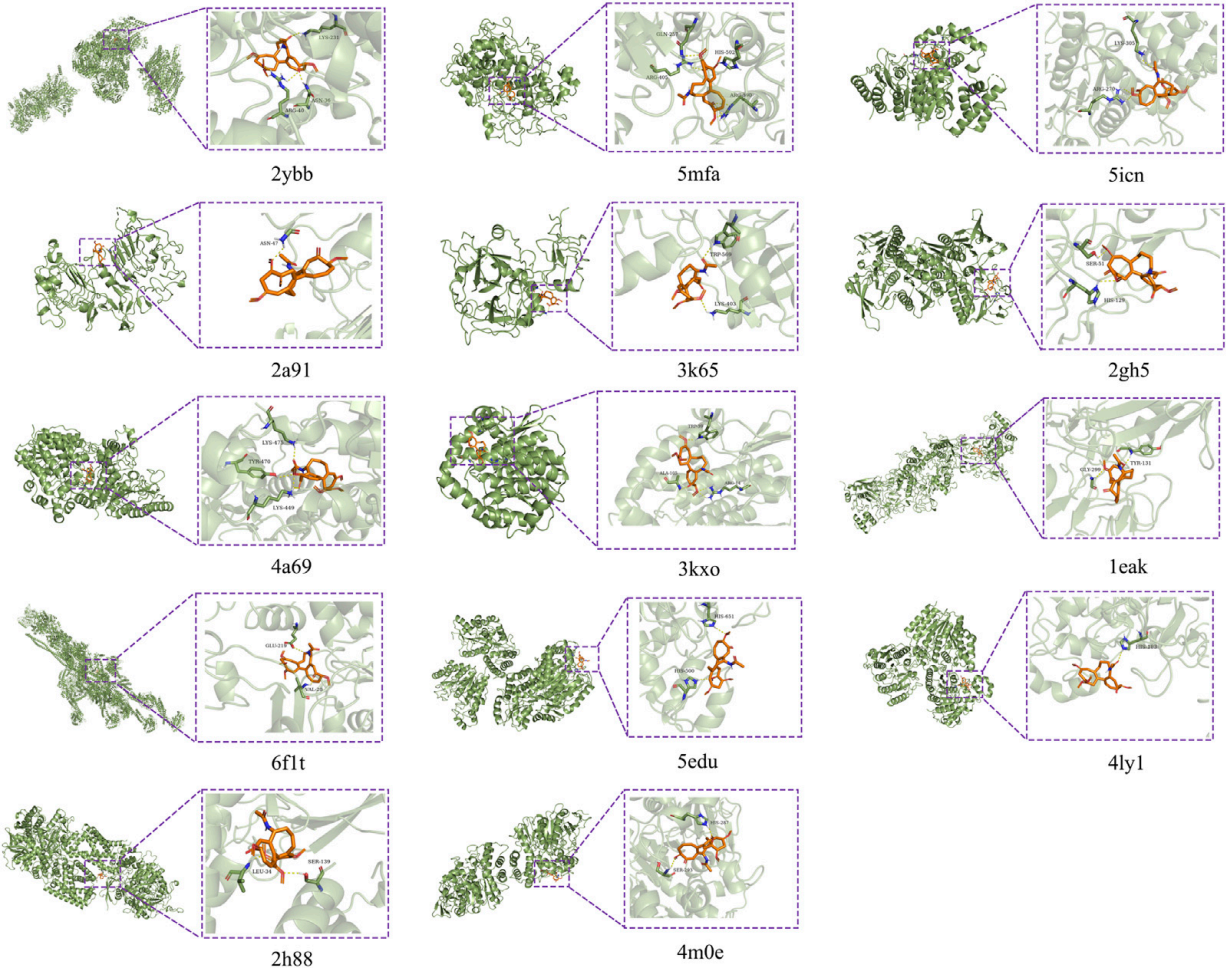
Molecular docking diagram.

Based on the results of the PPI, key targets with degree values > 5 were molecularly docked to the active ingredient colchicine. The stability between the two can be judged by the binding energy, which can be used to determine how well a protein binds to a ligand compound. When the binding energy of the ligand to the receptor is lower, the more stable the binding conformation of the two and the higher the possibility of interaction, which is generally evaluated at −5 kcal/mol (Liu et al., 2020; Lu and Wang, 2021). The results demonstrated that colchicine had a relatively high binding potential to CYCS, MPO, HDAC1, ERBB2, F2, GSR, HDAC3, HPGDS, MMP2, ACTA1, HDAC6, HDAC2, SDHA and ACHE (Table 1). Among them, the structure of CYCS protein is complex and PLIP cannot obtain the docking state of protein and ligand, so the molecular docking pattern of CYCS was not mapped. Molecular docking mode diagram is shown in Figure 7.

**TABLE 1:** Virtual docking of key targets to active ingredients.

Targets	PDB ID	Compound	Mol ID	Combined energy kcal/mol
CYCS	2 ybb	Colchicine	MOL013158	−6.7
MPO	5 mfa	Colchicine	MOL013158	−8.7
ANXA5	1 anx	Colchicine	MOL013158	−4.5
HDAC1	5 icn	Colchicine	MOL013158	−6.9
ERBB2	2 a91	Colchicine	MOL013158	−5.0
F2	3 k65	Colchicine	MOL013158	−6.8
GSR	2 gh5	Colchicine	MOL013158	−7.4
HDAC3	4 a69	Colchicine	MOL013158	−8.5
SOD2	1 pL4	Colchicine	MOL013158	191.4
HPGDS	3 kxo	Colchicine	MOL013158	−6.2
MMP2	1 eak	Colchicine	MOL013158	−6.7
ACTA1	6 f1t	Colchicine	MOL013158	−7.2
HDAC6	5 edu	Colchicine	MOL013158	−6.5
HDAC2	4 ly1	Colchicine	MOL013158	−6.6
SDHA	2 h88	Colchicine	MOL013158	−6.9
ACHE	4 m0e	Colchicine	MOL013158	−7.0

## Discussion

Target prediction yielded a total of 70 targets for colchicine in the treatment of CAD. PPI network further screened 4 key targets with degree values > 10 and 12 key targets with degree values > 5, which may be potential mechanism of colchicine in the treatment of CAD. Both MPO and CYCS are key targets with the highest degree values, and there are numerous studies demonstrating the involvement of MPO in the development and progression of atherosclerosis (Teng et al., 2017). The lack of MPO expression was shown to significantly reduce the risk of cardiovascular disease at the beginning of this century (Kutter et al., 2000), and subsequent studies have pointed out that MPO gene polymorphisms are associated with an increased risk of CAD (Asselbergs et al., 2004). A recent study suggested that elevated plasma MPO in patients with CAD were directly related to the severity of CAD and the risk of developing MACE, and that reducing MPO has important clinical implications for improving prognosis (Cheng et al., 2020). MPO has been reported to contribute to the development of CAD through multiple pathways, including triggering endothelial dysfunction, activation of inflammatory responses and weakening of atherosclerotic plaques (Chaikijurajai and Tang, 2020). Notably, it was reported that MPO can affect nitric oxide (NO) metabolism in the vascular endothelium by reducing NO production and inhibiting Bcl-2 expression, triggering apoptosis of endothelial cells and leading to vascular endothelial dysfunction (Kimak et al., 2018). And colchicine promotes NO production and exerts a protective endothelial function, probably by inhibiting the MPO pathway (Chen et al., 2022). Furthermore, it has been shown that MPO is also involved in the formation of neutrophil extracellular traps (NETs), which can activate the inflammatory response, promote the expression of MPO itself and its associated inflammatory factors, release matrix metalloproteinases and oxidants, and promote the formation, destabilization, as well as rupture of atherosclerotic plaques (Borissoff et al., 2013; Döring et al., 2017). Colchicine has been reported to reduce neutrophil L-selectin expression and regulate E-selectin expression on the cell surface of endothelial cells, thereby impairing neutrophil chemotaxis and adhesion, inhibiting superoxide production and expression of NETs, and attenuating cardiovascular injury from MPO (Tong et al., 2020). Besides, MPO binding to extracellular matrix structural proteins such as type IV collagen and fibronectin, which means that HOCl derived from MPO directly oxidizes the protein, can reduce the adhesion of vascular smooth muscle cells and affect atheromatous plaque stability (Kubala et al., 2013; Bennett et al., 2016). Thus, MPO is an important factor in the development and progression of atherosclerosis (Ndrepepa, 2019).

CYCS is the gene encoding cytochrome C, which is a water-soluble protein located in the inner mitochondrial membrane and is the only peripheral protein in the electron transport chain that plays an important role in mitochondrial energy metabolism (Marenzi et al., 2016). Continuous progressive damage to mitochondria is an important mechanism for the development of myocardial ischemia, which proceeds in a regional subpopulation-dependent manner and at specific sites in the electron transport chain within both groups of mitochondria (Lesnefsky et al., 2017). Because CYCS is located in the inner membrane of mitochondria, it is not detectable from the blood of healthy individuals under physiological conditions (Ow et al., 2008). However, prolonged ischemic injury can cause mitochondrial dysfunction, which in turn leads to CYCS release from mitochondria (Zhu et al., 2018). Colchicine can reduce the expression of NLRP3 inflammatory vesicles in mice with viral myocarditis and inhibit the expression of the apoptotic factor caspase-3, thereby reducing cardiac cell death (Pappritz et al., 2022). Colchicine gel was reported to inhibit myocardial apoptosis and fibrosis in mice with myocardial infarction and improved cardiac function and structure (Chen et al., 2020). Colchicine exerts its anti-inflammatory, anti-apoptotic and anti-fibrotic effects through upregulated hepatic B-cell lymphoma 2 (Bcl-2) and downregulated Bcl-2 associated X protein (BAX) expression and transforming growth factor-β (TGF-β) content, which attenuates rat liver injury induced by renal ischemia-reperfusion injury (Awad et al., 2022). It was shown that when cytochrome C is released from mitochondria, it activates the caspase-3 apoptotic pathway and promotes cardiomyocyte apoptosis, making cardiac function impaired (Radhakrishnan et al., 2007). In summary, colchicine has an inhibitory, anti-apoptotic and anti-fibrotic effect on cardiomyocyte inflammation. Thus, we speculate that colchicine may maintain mitochondrial integrity and reduce the release of cytochrome C after myocardial damage through its inhibitory effect on cardiomyocyte apoptosis. And as an important substance affecting mitochondrial function, CYCS is then likely to be a key target for the treatment of CAD.

ANXA5, also known as membrane-linked protein A5, is a member of the Annexin family (Zhang et al., 2019a). It inhibits the binding and uptake of oxidized LDL by macrophages and plays an important protective role in atherosclerosis formation and plaque rupture (Domeij et al., 2013). Myocardial infarction leads to elevated levels of endogenous ANXA5, which is a self-protective mechanism of the body (Matsuda et al., 2003). Treatment with exogenous ANXA5 reveals that labeled ANXA5 accumulates in a punctate pattern in the infarcted area, which is significantly reduced in the infarcted area of myocardial infarction after 3 weeks (de Jong et al., 2018). The mechanism is the reduced proliferation of macrophages and inhibition of IL-6 production by macrophages derived from bone marrow, attenuating the inflammatory response (de Jong et al., 2018). ANXA5 also significantly inhibits the capture, rolling, adhesion and migration of peripheral blood monocytes on TNF-α-activated endothelial cell layer, which may reduce inflammation of plaques in lesions with advanced disease by interfering with the recruitment and activation of monocytes at the site of inflammatory lesions (Burgmaier et al., 2014). It was noted that colchicine not only binds microtubulin heterodimers to alter the conformation of microtubulin and prevent microtubule growth, thus affecting the assembly of inflammatory vesicles, but also inhibits neutrophil chemotaxis and reduces macrophage formation, thus exerting anti-inflammatory effects (Slobodnick et al., 2015). ANXA5 has also been reported to further stabilize plaques by reducing the content of macrophages and increasing the content of smooth muscle cells (Stöhr et al., 2017). They also suggest that ANXA5 treatment reduces the formation of atherosclerotic plaques, in part due to a reduced rate of apoptosis, thus favoring macrophage infiltration and activation (Stöhr et al., 2017). Colchicine can inhibit lipoprotein uptake by macrophages and is also able to suppress formation of foam cell by promoting lipid efflux from macrophage ABCA1 receptors (Zhang et al., 2022). It has been suggested that ANXA5 enhances ABCA1-mediated cholesterol efflux, thereby improving macrophage lipid metabolism and reducing the risk of CAD (Chen et al., 2021). However, the role of ANXA5 in CAD remains controversial. Burgmaier et al. revealed that in high-risk diabetic patients, the level of circulating ANXA5 correlated with the thickness of the carotid intima, but not with the composition of the coronary plaque (Burgmaier et al., 2017). And Hashemi et al. reported that the frequency of polymorphism in ANXA5 gene was not relevant to the presence of in-stent restenosis (Hashemi et al., 2019). Nevertheless, the anti-atherosclerotic potential of ANXA5 still deserves further attention and research, and ANXA5 may be an important target for the treatment of CAD.

**FIGURE 8:**
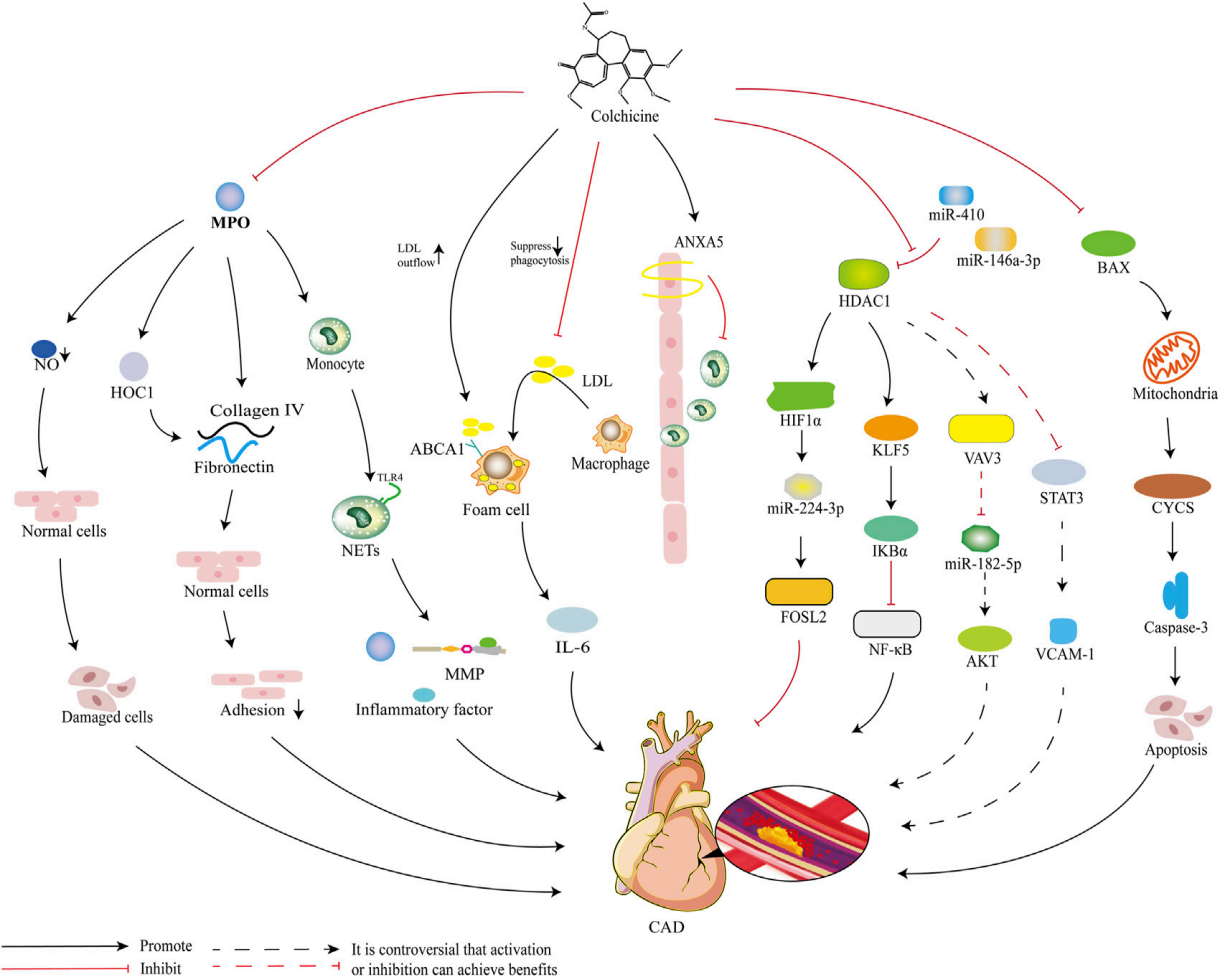
Diagram of the potential mechanism of colchicine for the treatment of CAD.

HDAC1, histone deacetylase 1, is an enzyme that removes acetyl groups from lysine residues of histones and non-histones (Pao et al., 2020). HDAC1 has a protective role in models of atherosclerosis and is able to mediate the effects of external and ambient stimuli by modulating key endothelial functions such as angiogenesis, inflammatory signaling, redox homeostasis and nitric oxide signaling (Dunaway and Pollock, 2021). The anti-atherosclerotic effect of HDAC1 may be related to the miR-410/HDAC1/KLF5/IKBα/NF-κB axis, with miR-410 playing a potential role in the pathogenesis of atherosclerosis (Nan et al., 2021). Studies have suggested that blocking miR-410 promotes HDAC1 expression, increases KLF5-mediated IKBα expression, and inhibits the NF-κB pathway, thereby inhibiting the development of atherosclerosis (Nan et al., 2021). It has also been demonstrated that the anti-atherosclerotic effect of HDAC1 is based on the miR-146a-3p/HDAC1/KLF5/IKBα signaling axis, as deletion of miR-146a-3p promotes the expression of HDAC1 and IKBα in atherosclerotic mice, thus promoting the stabilization of pathological plaques (Liu et al., 2021). It has also been reported that the role of HDAC1 is closely related to miR-224-3p and that the over expression of HDAC1 may enhance the anti-atherogenic and endothelial protective effects of miR-224-3p-mediated FOSL2 inhibition by deacetylating HIF1α (Wang et al., 2020). Although there is more evidence that HDAC1 plays a protective role in atherosclerosis and CAD, there is still evidence supporting HDAC1 as an adverse factor in atherosclerosis. It has been noted that HDAC1 can be involved in VCAM-1 expression and promotion of atherosclerosis through inhibition of STAT3 acetylation-dependent methylation of the GATA6 promoter (Hu et al., 2021). It has also been proposed that HDAC1 inhibits miR-182-5p and activates the AKT pathway by improving VAV3, thereby promoting the progression of atherosclerosis (Gao et al., 2021). Furthermore, some reports have demonstrated that high expression of HDAC1 suppresses H3K9ac and promotes the accumulation of total cholesterol, free cholesterol and triglycerides in foam cells (Zhao et al., 2017). All of these factors may contribute to the progression of atherosclerosis and CAD. Although the beneficial and detrimental effects of HDAC1 in atherosclerosis and CAD remain controversial, the important value of HDAC1 in atherosclerosis and CAD cannot be denied. What is certain is that HDAC1 regulates endothelial function through deacetylation of histones and non-histones, which ultimately affects overall physiology and health. The potential mechanism of colchicine in treating CAD is shown in Figure 8.

In molecular docking, colchicine has a high probability of binding to three key targets with degree >10, such as MPO, CYCS and HDAC1, and other 11 key targets with degree >5. MPO is the key target with the highest degree value and lowest binding energy, and the binding energy for docking with colchicine is −8.7 kcal/mol, which is a good fit. Studies have revealed that colchicine can significantly reduce MPO, and this effect has been demonstrated in models of respiratory syncytial virus infection (Lu et al., 2019), ischemia-reperfusion injury in allogeneic lung transplants (Pieróg et al., 2007), and ischemia-reperfusion-induced injury to skeletal muscle (Wang et al., 2016). CYCS is the key target ranked second in terms of degree value, with a binding energy of −6.7 kcal/mol for docking with colchicine and a good fit. Available studies have shown that colchicine promotes the release of CYCS to activate apoptosis in acute lymphoblastic leukemia (Aghvami et al., 2018), as well as the release of CYCS to induce apoptosis in gastric cancer cells and inhibit growth of gastric tumors (Zhang et al., 2019b). It has also been reported that after pretreatment of HPV16-positive and HPV18-positive human cervical cancer cell lines with colchicine for 48 h, there was a significant increase in CYCS expression in the cytoplasm (Yan et al., 2020). This implies that colchicine is a promising candidate for the treatment and prevention of HPV-associated cervical cancer (López-Sánchez and Frade, 2002). ANXA5 is the key target ranked third in degree value, but the binding energy of ANXA5 docked with colchicine is −4.5 kcal/mol, suggesting that the binding of both is less likely. HDAC1 is the last key target with a degree value > 10 and has a good binding energy of −6.9 kcal/mol docked with colchicine, which is a good fit. Yet, the effect of colchicine on HDAC1 has not been reported in the literature and is to be demonstrated by relevant studies.

In the KEGG pathway enrichment analysis, cellular response to chemical stress and p75NTR-mediated SC1 negative regulation of cell cycle may be the main pathways for colchicine to treat CAD. The cellular response to chemical stress is the top-ranked signaling pathway, and CYCS, HDAC3, GSR, SOD2, SIN3B, NCOA2, PPARA, and BLVRA are all intersectional targets enriched in this pathway. Among them, CYCS and HDAC3 are key targets with degree >10, and GSR, SOD2 and SIN3B are the key targets with degree >5. The p75NTR-mediated SC1 negative regulation of cell cycle is the second-ranked signaling pathway, and three key targets, HDAC1, HDAC2, and HDAC3, are enriched in this pathway. They cover the entire range of histone deacetylases in this process and play an integral and important role in mediating the deacetylation of PRDM4 in this pathway (López-Sánchez and Frade, 2002). In GO functional enrichment analysis, the cellular component of colchicine for the treatment of CAD is mainly the nucleus, while the molecular function mainly affects protein binding and the biological process mainly affects metabolic processes. These are consistent with the results of current studies. It has been reported that colchicine inhibits DNA release by inhibiting the formation of neutrophil-spontaneous, foponol-12-myristate-13-acetate-induced and ionomycin-induced reticulation in patients with acute coronary syndrome, which immobilizes the neutrophil microtubule complex around the nucleus (Vaidya et al., 2021).

Although this study predicted potential targets and mechanisms for colchicine in the treatment of CAD, it still has some limitations (shown below). 1) Due to the limitations of the databases, some targets and mechanisms may not have been included in the analysis. Deftereos et al. showed that colchicine can reduce the incidence of in-stent restenosis by inhibiting the in-stent neointima (Deftereos et al., 2013), and Saji et al. found that colchicine can inhibit cardiomyocyte apoptosis by inhibiting the Bax/Bcl-2 pathway (Saji et al., 2007). Manni et al. also found that nerve growth factor (NGF) and brain-derived neurotrophic factor (BDNF) may be related to the pathogenesis of CAD, and the changes in the levels of these two neurotrophins are related to inflammation, so NGF and BDNF may also be potential targets for colchicine in the treatment of CAD (Manni et al., 2005). In addition, the classic anti-inflammatory mechanism of colchicine is to inhibit tubulin polymerization, which means that tubulin-related targets may be the entry point for future research (Chaldakov, 2018). These mechanisms appear to be beneficial for CAD, but this study did not predict them. 2) Network pharmacology and molecular docking can only be used to predict the targets of colchicine for the treatment of CAD, but not for its efficacy and safety assessment. The long-term safety of colchicine in the treatment of CAD is still unclear and this is to be explored in ongoing clinical trials. In fact, the side effects of a drug are closely related to its selectivity, and the broader the range of action of colchicine in the body means that its potential side effects may be stronger. Therefore, some researchers have focused their attention on new colchicine formulations. Eddleston et al. reported that equimolar doses of anti-colchicine Fab given early in life reduced the cardiotoxicity of colchicine (Eddleston et al., 2018). And Chen et al. used an injectable thermosensitive polymer hydrogel as a carrier for colchicine in the treatment of myocardial infarction, which reduced the systemic toxicity of colchicine (Chen et al., 2020). This implies that colchicine preparations acting topically may have a higher safety profile, and this may be a future research direction.

## Conclusion

This study predicts the potential targets and mechanisms of action of colchicine in the treatment of CAD. Colchicine may treat CAD through targets such as CYCS, MPO and HDAC1. The mechanism of action may be related to the cellular response to chemical stimulus and p75NTR-mediated negative regulation of cell cycle by SC1, which is valuable for further research exploration. However, our research lacks experimental verification, and we look forward to future experimental studies to confirm our results and continue to explore new drugs for treating CAD on this basis.

## Data Availability

The original contributions presented in the study are included in the article/Supplementary Material. Further inquiries can be directed to the corresponding author.
